# Dexamethasone and IFN-γ primed mesenchymal stem cells conditioned media immunomodulates aberrant NETosis in SLE via PGE2 and IDO

**DOI:** 10.3389/fimmu.2024.1461841

**Published:** 2024-10-31

**Authors:** Khushbu Priya, Hiral Thacker, Manaswi Chaubey, Madhukar Rai, Shambhavi Singh, Sonali Rawat, Kiran Giri, Sujata Mohanty, Geeta Rai

**Affiliations:** ^1^ Department of Molecular and Human Genetics, Institute of Science, Banaras Hindu University, Varanasi, India; ^2^ Department of Medicine, Institute of Medical Sciences, Banaras Hindu University, Varanasi, India; ^3^ School of Medicine, DY Patil University, Navi Mumbai, India; ^4^ Stem Cell Facility, DBT-Centre of Excellence for Stem Cell Research, AIIMS, New Delhi, India; ^5^ Department of Pharmacology, Institute of Medical Sciences, Banaras Hindu University, Varanasi, India

**Keywords:** NETosis, neutrophil extracellular trap formation, systemic lupus erythematosus, reactive oxygen species, hydroxychloroquine, ribonucleoprotein immune complexes, lupus model

## Abstract

**Background:**

Systemic Lupus Erythematosus (SLE) is characterized by dysregulated immune responses, with neutrophil extracellular traps (NETs) playing a significant role. NETs are recognized by autoantibodies in SLE patients, exacerbating pathology. Both excessive NET formation and impaired degradation contribute to SLE pathophysiology.

**Objective:**

To investigate the immunomodulatory effects of Dexamethasone-primed Wharton’s jelly (WJ) derived MSCs CM (DW) and IFN-γ-primed WJ-MSCs-CM (IW) on NETosis and associated protein markers in SLE patients’ LPS or ribonucleoprotein immune complexes (RNP ICs) induced neutrophils and in pristane induced lupus (PIL) model. And to elucidate the mechanism involved therein.

**Methods:**

We investigated the immunomodulatory effects of DW and IW on NETosis in SLE. Utilizing *ex vivo* and *in vivo* models, we assessed the impact of preconditioned media on NET formation and associated protein markers neutrophil elastase (NE), citrullinated histone (citH3), myeloperoxidase (MPO), cytoplasmic and mitochondrial ROS production. We also examined the involvement of key immunomodulatory factors present in DW and IW, including prostaglandin E2 (PGE2), indoleamine 2,3-dioxygenase (IDO), and transforming growth factor-beta (TGF-β).

**Results:**

Preconditioned media effectively suppressed NETosis and reduced ROS generation in SLE neutrophils, indicating their immunomodulatory potential. Inhibition studies implicated IDO and PGE2 in mediating this effect. Combined treatment with DW or IW together with hydroxychloroquine (HCQ) demonstrated superior efficacy over HCQ alone, a standard SLE medication. In PIL mouse model, DW and IW treatments reduced NETosis, ROS generation, as evidenced by decreased NET-associated protein expression in vital organs.

**Conclusion:**

Our study highlights the multifaceted impact of IW and DW on NETosis, ROS dynamics, and lupus severity in SLE. These findings underscore the potential of preconditioned media for the development of targeted, personalized approaches for SLE treatment.

## 1 Introduction

Systemic Lupus Erythematosus is a complex autoimmune disease characterized by dysregulated immune responses, aberrant cell death pathways, and the generation of autoantibodies against self-antigens (1). Among the myriad immunological aberrations in SLE, the role of neutrophil extracellular traps (NETs) and reactive oxygen species (ROS) has emerged as crucial contributors to the disease pathogenesis (2). NETosis, a vital antimicrobial mechanism employed by neutrophils, involves the expulsion of decondensed chromatin and granular contents into the extracellular space, contributing to the immunogenicity of dsDNA and citrullinated autoantigens in SLE with heightened autoimmune responses and organ damage. In contrast to apoptotic cells, netting neutrophils do not appear to display eat me signals, and this may prevent their clearance by phagocytes (3). Neutrophils in lupus patients are characterized by increased capacity to undergo NETosis, hence NETosis is considered an important source of citrullinated autoantigens and immunogenic dsDNA in SLE (4). Concurrently, the aberrant production of ROS, particularly within neutrophils, further amplifies the inflammatory milieu and sustains the NETotic process. Autoantibodies such as anti-dsDNA and anti-RNP are hallmarks of SLE, and their prevalence is linked to dysregulated NETosis (5).

The accumulation of neutrophils, monocytes, and immune complexes in tissues, coupled with dysregulated B and T cell responses, further contributes to the autoimmune pathology in SLE (6). Wharton jelly derived mesenchymal stem cells (WJ-MSCs) are known for their remarkable immunomodulatory and anti-inflammatory properties, making them promising candidates for autoimmune disease therapy. Stem cell derived secretory factors or the secretome collected from the culture media is referred as cell free conditioned media (CM) and has several advantages over the use of MSCs. Further, preconditioning the MSCs prior to preparing the CM (pre-conditioned CM) enhance the ability of the secretome to regulate both the innate and adaptive immune responses in patients affected by immune system related diseases (7–9), hence, this approach can be explored for immunomodulating NETosis in SLE.

IFN-γ or Dexamethasone preconditioned MSCs CM have not yet been examined, despite prior attempts to investigate the immunomodulatory effects of preconditioned media employing phorbol esters (10), poly-inosinic acid/cytidylic acid (11), and others in SLE. Dexamethasone-primed Wharton’s jelly (WJ) derived MSCs CM (DW) and IFN-γ-primed WJ-MSCs-CM (IW) in particular attracted a lot of attention because they could reduce the usage of glucocorticoids and IFN-γ, respectively, which are known to cause morbidity because of their adverse effects. Therefore, such an approach represents an attractive strategy for the treatment of SLE. Patients may have a higher quality of life and lower overall rates of morbidity by using glucocorticoids less frequently.

Dexamethasone is known for its role in the augmentation of the anti-inflammatory effects and immunosuppressant properties. Dexamethasone is a synthetic glucocorticoid having shown promising results in diseases like arthritis, cancer, asthma, COVID 19 (12). IFN-γ also plays a crucial role in the self-regulation of inflammatory responses, serving both as an initiator and a controller of inflammation. Hence, we used IFN- γ and Dexamethasone preconditioned CM in this study anticipating their potential prospect of a promising immunomodulator for NETosis in SLE. In a parallel lab study using DW, a significant expansion of Tregs and Bregs, suppression of Th17, double-negative T cells, and inflammatory neutrophils through modulating IL-10 and IL-17A production and attenuating autoantibody production, increased anti-inflammatory cytokines, and maintenance of a balanced Th17/Treg ratio were observed. In the preclinical *in vivo* investigations, DW demonstrates therapeutic efficacy, improving disease pathology, lowering mortality, preventing proteinuria, and reversing limb inflammation, seizures, alopecia in the mice lupus model. The kidneys, liver, lungs, heart, and spleen were all protected by DW.

Hence in this study, we present the results of comprehensive elucidation of the impact of Dexamethasone, DW, IW and W on NETosis and ROS dynamics in SLE, utilizing both ex vivo experiments with patient-derived neutrophils and *in vivo* PIL mouse model. Importantly, our research builds on existing literature that highlights the intricate relationship between NET formation, chromatin decondensation, and oxidative stress in the context of SLE pathophysiology. Our investigations encompassed a diverse array of experimental approaches, including the evaluation of NETosis in neutrophils isolated from SLE patients, assessments of ROS generation, and the utilization of contrast agents for *in vivo* imaging to ascertain the therapeutic potential of DW and IW. Furthermore, we probed the immunomodulatory mechanisms underlying the effects of DW and IW by exploring the involvement of key factors secreted by WJ-MSCs, including prostaglandin E2 (PGE2), indoleamine 2,3-dioxygenase (IDO), and transforming growth factor-beta (TGF-β). Excessive NET formation and its impaired degradation are implicated in SLE pathophysiology, contributing to elevated autoantibodies.

This comprehensive study elucidates the NETotic responses in SLE, with a focus on the immunomodulatory potential of DW and IW. The findings underscore the promising therapeutic role of DW and IW, alone or in combination with Hydroxychloroquine (HCQ), in regulating NETosis and ROS generation, offering potential avenues for future SLE treatment strategies.

## 2 Materials and methods

### 2.1 Subjects, sample collection, and clinical assessment

The study subjects were recruited from Sir Sunderlal Hospital, Banaras Hindu University (BHU), Varanasi, Uttar Pradesh, India. Ethical clearance was obtained from the Institutional Ethics Committee of the Institute of Medical Science, Banaras Hindu University (Ref. Dean/2020/EC/Ro32, Dated: 27.06.2020) for the collection of samples from SLE patients (n=42) and healthy adults (n=3) after obtaining written informed consent for participation in this study. Detailed information regarding age, sex, medical history, symptoms, medications, and clinical manifestations was collected on a predesigned proforma. The inclusion criteria for this study were as follows: age ≥ 18 years, SLE or suspected SLE established by American College of Rheumatology (ACR) criteria, and glomerulonephritis and pericarditis. The exclusion criteria were as follows: history of hepatitis B or C, history of HIV, cancer, pregnancy or lactation, diagnosis of diabetes and/or HbA1C level >6%, and any comorbidity of medical, psychological/psychiatric condition, or treatment.

#### 2.1.1 Isolation of neutrophils

Neutrophils were isolated from the peripheral blood of both SLE patients and healthy adults by using a density gradient method. The mean purity of neutrophils were at least 94%. To accomplish this, the blood was centrifuged on HiSep and GranuloSep (Himedia, India) and the resulting lower phase, which was composed mostly of granulocytes, was treated with RBCs lysis buffer. Afterwards, the sample was washed with PBS and supplemented with 10% low-endotoxin fetal bovine serum in RPMI 1640.

#### 2.1.2 Immunomodulatory impact of DW and IW on neutrophils

Isolated neutrophils (1×10^6^ cells/well) were induced with TLR-4 agonist LPS 1 µg/mL or uninduced and incubated with 40% non-preconditioned MSC-derived conditioned media (W) or 40% Dexamethasone drug (7.6 µM), DW, IW (4.76 nM) for 24 h.

#### 2.1.3 Determination of anti-RNP ICs concentration used for NETosis induction

Neutrophils (1×104 cells/well), stimulated with serum isolated from SLE patient containing high titer of anti-RNP ICs (47 U/ml) and anti-Sm (5.9 U/ml) antibodies at a final volume of 10, 20 and 40 µl, were cultured in 96-well plates as described above for 24 h.

#### 2.1.4 Immunomodulatory impact of DW and IW on SLE patient’s neutrophils

After determination of optimum concentration that can be used for induction of NETosis and generation of mitochondrial as well as cytoplasmic ROS, neutrophils (1×104 cells/well) were stimulated with 10 µl of serum having high titer of anti-RNP ICs (47 U/ml) and anti-Sm (5.9 U/ml) antibodies and treated with 40% Dexamethasone (7.6 µM), DW, IW and W for 24 h.

#### 2.1.5 Comparison of immunomodulatory impact of DW, IW and HCQ

To compare the immunomodulatory effect of DW and IW with standard drug HCQ and the impact of their combination, 1×10^6^ neutrophils unstimulated or stimulated with RNP ICs positive SLE patient serum (10 µl) were incubated with 7.6 µM HCQ (Sigma-Aldrich), Dexamethasone drug (7.6 µM), DW, IW (4.76 nM) and W for 24 h and stimulated as described above.

#### 2.1.6 Inhibition study

SB 431542 hydrate (Sigma Aldrich), NS398 and 1MT were used as an inhibitor of TGF-β, PGE2 and IDO activity respectively to study the functional relevance of TGF-β, PGE2 and IDO in DW/IW mediated suppression of NETosis. For this, neutrophils were incubated with SB 431542 hydrate (Sigma Aldrich) + NS398 (Cayman), NS398 + 1MT (Sigma Aldrich) or 1MT + SB 431542 hydrate each at a concentration of 5 µM prior to addition of Dexamethasone drug, DW, IW and W incubated for 24 hrs.

### 2.2 Animals

Female BALB/c mice (n=42, aged 8 weeks, weighing 20 ± 2 g) were sourced from the Institute of Medical Science, BHU (Varanasi, India). These mice were kept in a barrier system under a 12-hour light/dark cycle, with a relative humidity of 50 ± 5% and a constant temperature of 25°C. The study protocols involving the animals were reviewed and approved by the IAEC of the Institute of Science, BHU, India (Ref no.: BHU/DoZ/IAEC/2022-2023/003).

#### 2.2.1 Development of PIL model

The mice (n=35) received an intraperitoneal injection of pristane (0.5 ml) (Sigma-Aldrich) for disease development. A control group of healthy mice (n=7) received normal saline (1.3 ml) for 6 weeks. Following 4 weeks of pristane treatment, the development of a PIL model was assessed by evaluating lupus-like pathology: presence of autoantibodies like anti-dsDNA, anti-ENA and high proteinuria.

For detection of anti-dsDNA and anti-ENA autoantibodies post pristane injection, blood was collected from the tail vein at 4 weeks, and serum was isolated. Using ELISA kits (MyBioSource) the serum levels of IgG autoantibodies against dsDNA and ENA were measured following the manufacturer’s instructions. Urine was collected on 35th day from the control, and the four PIL groups of mice in the morning into a clean microcentrifuge tube. Urine was dropped into a urine analysis strip (Uristix, Siemen) and analyzed according to the manufacturer’s instructions.

The PIL mice were then divided into five groups (n=7 per group) and administered intraperitoneal injections of normal saline, Dexamethasone drug, DW and W daily for the next 6 weeks. The treatment groups were as follows: group 1 (No treatment Lupus group) received normal saline (1.3 ml); group 2 received Dexamethasone drug; group 3 received DW; group 4 received IW (4.76 nM) subcutaneously on alternate days; and group 5 received W.

#### 2.2.2 Mouse neutrophil purification

Circulating neutrophils were purified from healthy and PIL mice on various treatments. Retro orbital blood was collected by laterally inserting a sterile capillary tube in the retro orbital sinus of the eyes, and blood was allowed to flow by capillary action into the heparinized (Sigma) centrifuge tube via capillary tube. The blood was diluted with an equal volume of PBS and subjected to a discontinuous Histopaque (Sigma) gradient (1.077 and 1.119). Neutrophils were collected from the interface. Red blood cells were eliminated by RBC lysis. Neutrophil purity and viability were determined visually and were consistently >98%.

At week 42 (6 weeks following treatment), euthanasia was carried out using an approved method to minimize suffering, and organs were harvested to assess the effect of Dexamethasone drug, preconditioned, and non-preconditioned media. The mice were placed in a supine position for optimal access to abdominal organs. Abdominal organs, including the lungs, heart and kidneys were dissected using sterile surgical instruments. Each organ was placed in a clean labelled Petri dish. Further, tissue section was dissected and stained for detection of NETosis (citH3 and NE).

#### 2.2.3 Visualization of NETs by fluorescence microscopy

Neutrophils (1×10^6^ cells/ml) from SLE patients were seeded onto 0.001% poly-L-lysine coated glass coverslips and left uninduced or induced with LPS and treated with 40% Dexamethasone (7.6 µM), DW, IW and W for 24 h at 37°C in 5% CO2. Afterward, the cells were washed and stained with Syto 13 (green) and Sytox orange (red) at 0.5 µM final concentration to label intracellular and extracellular DNA, respectively. The cells and tissue sections from mice organs were then fixed with 4% paraformaldehyde for 30 min, washed with PBS twice, permeabilized with 0.1% Triton-X for 5 minutes, and blocked with 5% BSA in PBST for an hour. Next, the cells were incubated overnight with primary antibodies mouse anti-human NE [1:100] (Santa Cruz Biotechnology) and rabbit anti-human CitH3 [1:250] (Abcam) in PBST containing 5% BSA. After washing, the cells were incubated with TRITC conjugated goat anti-mouse IgG and FITC conjugated goat anti-rabbit lgG secondary antibodies [1:500] and [1:400], respectively, diluted with 5% BSA in PBST. Finally, the cells were stained with DAPI (1 µg/mL) (Sigma Aldrich) for 20 min and mounted with DABCO (Sigma-Aldrich) before being examined using a confocal laser scanning microscope system (Zeiss LSM780, Carl Zeiss) and processed using Zen lite 2012 (Carl Zeiss).

#### 2.2.4 Extracellular DNA assay

Neutrophil (1×104 cells/ml) were seeded in a 96-well black plate (Cayman Chemical) and were treated with Dexamethasone, DW, IW, and W drugs for 24 h. To detect NETs in the culture supernatants, 100 µL of the supernatant from each well was transferred to another well. The amount of dsDNA in the culture supernatants was measured by adding Sytox Orange at a concentration of 0.5 µM to bind extracellular DNA, and fluorescence was quantified using a SynergyTM MxBioTek multimode microplate reader at 547 nm (excitation)/570 nm (emission) with Gen5 2.01.14 software.

#### 2.2.5 Flow cytometry to detect ROS generation

To analyze the production of ROS in neutrophils using flow cytometry, we collected 1×10^6^ cells from each culture and suspended them in 0.5 ml of RPMI medium supplemented with 5 μM MitoSOX and 5 μM DCFDA, which were used to assess mitochondrial and cytoplasmic ROS production, respectively. This was done by incubating the cells in an Eppendorf tube at 4°C in the dark for 20 min. Following this, the cells were gently washed three times with prewarmed PBS at 37°C, mixed gently after each wash, and then resuspended in PBS for detection. The flow cytometer (Beckman Coulter CytoFLEX) was used to acquire the cells, and the mean fluorescence intensity and percentage of stained cells were measured. The results were analyzed using CytExpert software (Beckman Coulter) and expressed as a histogram of the cell count of cell fluorescence emission, representing the size of the cell population emitting the corresponding fluorescence.

#### 2.2.6 Quantification of ROS

The generation of ROS was determined using 2,7-Dichlorofluorescin diacetate (DCFDA) (Sigma-Aldrich) and MitoSox Red dye (Molecular Probes). DCFDA is a non-fluorescent, cell-permeable dye that displays green fluorescence upon oxidation, making it ideal for quantifying cytosolic ROS. MitoSox Red dye is also permeable to live cells and is rapidly and selectively targeted to the mitochondria, it is oxidized by superoxide, resulting in red fluorescence once inside the mitochondria. Neutrophils (1×104 cells/ml) were seeded into 96-well plates and were incubated with Dexamethasone drug, DW, IW, and W for 24 h. After incubation with DCFDA (0.5 µM) and MitoSOX Red dyes (0.5 µM), the signals were quantified using a multi-mode microplate reader (SynergyTM MxBioTek) and analysed with Gen5 2.01.14 software. The excitation and emission wavelengths for DCFDA were 504 nm and 530 nm, respectively, and for MitoSOX Red, they were 510 nm and 580 nm.

#### 2.2.7 Evaluation of cytotoxicity

To assess whether the Dexamethasone drug, DW, IW, W, and anti-RNP-ICs, anti-Sm antibodies present in SLE patient serum have any cytotoxic effect on neutrophils, the MTT (HiMedia) assay was performed using a concentration of 5 mg/ml. After adding MTT to the cells (1×104) for four h, 0.04M HCl isopropanol was added to the medium containing the MTT solution and incubated at 37°C for one hour in the dark. The absorbance was measured at 570 nm using a 96 well microplate reader (SynergyTM HT, Bio-Tek Instruments, Inc.).

#### 2.2.8 *In vivo* assessment of NETosis using PAI

We have used the ‘Phantom’ application of the Vevo LAZR-X system to assess the efficacy of photoacoustic contrast agents, such as the anti-MPO antibody and sytox orange, in soft-tissue mimicking phantoms. The system’s spectro mode was employed to analyze the absorption spectra of these agents, with the goal of determining their potential for use *in vivo* as optoacoustic agents.

High-resolution ultrasound imaging was conducted using a Vevo3100-LAZRX small animal PAI system, equipped with an MX-550D linear-array transducer (25-50 MHz, scan depth of 15 mm) for the abdominal region and a 250S transducer (15-30 MHz, scan depth of 30 mm) for the cardiac region. Imaging parameters were set as follows: PA gain = 37 dB, step size 0.279 mm, power 100%, and sensitivity was kept high. Abdominal hair was removed using a depilatory and hair remover cream, while alcohol pads were used to thoroughly clean the scalp. To cover the 5 mm gap between the transducer surface and mouse skin, a bubble-free, clear ultrasonic gel (OXD) was applied. The following imaging parameters were set for photoacoustic imaging: PA gain = 37 dB, step size of 0.279 mm, power at 100%, and sensitivity was kept high. During imaging, mice were anesthetized with 2% isoflurane (induction dose) and 1.5-2% maintenance dose. The animals were then placed in a supine position on an operating table maintained at 37°C for the ultrasound and photoacoustic imaging, with their body temperature, heart rate, and respiration rate monitored. One of the primary imaging methods used was B-mode imaging, which provided a real-time, cross-sectional view of the internal structure of the organs, such as the kidneys, liver, and heart. This mode also allowed for the visualization of organ shape, size, and position.

Prior to its *in vivo* application, the PA contrast agent’s efficacy was rigorously examined in optically diffusive phantoms, mimicking the optical properties of living tissue and providing insights into light-tissue interaction phenomena for fluorescence assessment. Polymeric blood vessel-mimicking tubes were employed as optically diffusive phantoms, resembling living tissue in optical properties and fluorochrome biodistribution. Phantoms contained cylindrical structures with fully submerged thin tubes, representing blood vessels, filled with Sytox Orange or MPO. A blank tube and another filled with water were included for comparison. The optical characteristics of the phantoms allowed the assessment of fluorescence signals and associated wavelengths for Sytox Orange and MPO. Targeted anti-MPO antibodies and Sytox Orange were diluted in normal saline to concentrations of 100 μg/ml and 1 nmol, respectively. These solutions were injected into the blood vessel-mimicking tubes, followed by PAI in combination with conventional ultrasound. The PA signal intensity was recorded in the range of 680 to 700 nm. The maximum photoacoustic intensity of MPO and Sytox Orange in the phantom capillary tubes was observed after laser irradiation at 680 nm (**Supplementary Figure 1**). This optimal wavelength was subsequently utilized to investigate the accumulation of these fluorochromes in the liver, kidneys, and heart of control, lupus, Dexamethasone drug, DW, IW and W treated mice.

For the study, mice were intra-peritoneally injected with normal saline, Dexamethasone drug, DW, IW, and W for six weeks prior to imaging. To assess NET protein markers, PE-labeled anti-MPO antibody (Beckman Coulter) at a concentration of 100 μg/ml in normal saline was administered for 30 minutes at 37°C. To examine extracellular DNA release, after 60 min of imaging, Sytox Orange (1 nmol/mouse i.v.) was administered and kept on a heated platform for 30 min before imaging, with the mice’s body temperature, heart rate, and respiration rate monitored. Images were then recorded and merged to produce single dual-color images of the liver, kidneys, and heart.

### 2.3 Statistical analysis

Statistical analyses were carried out using Prism (GraphPad version 10.1.2). The mean ± SEM of at least three independent experiments was calculated for all experiments. One-way ANOVA and two-way ANOVA were used to compare the means between two or more groups, followed by Tukey’s multiple comparison *post-hoc* test. Specific P values are detailed in the figure legends. Differences were considered significant when p-values were < 0.05.

## 3 Results

### 3.1 Subject characteristics

Among 42 SLE patients enrolled in this study 39 were females and 3 males, with ages range 18-55 years. **Table 1** depicts the distribution of SLE patients according to the presence of distinct clinical manifestations and medications under use.

**Table 1:** Patient details.

S.No.	Age	Gender	Symptoms	ANA	dsONA	ENA	Meditation
1.	22	F	Lupus nephritis, a utoimmune tiemolytic anemia, MCTO, seizure, heoatosolenomeealv	+ve	+ve		HCQ, Omnacortil, Nicardia
2.	18	F	SLE	+ve	+ve		HCQ
3.	25	F	SLE	+ve	+ve		HCQ
4.	19	M	SLE with autoimmune hemolytic anemia	+ve	+ve		HCQ, Omnacortil
5.	38	F	SLE	+ve	+ve	+ve	HCQ
6.	26	F	SLE, polyarticular pain	+ve	+ve	+ve	HCQ, Omnacortil
7.	27	F	SLE with upus Nephritis, MCTD, PSS, LTRI. Hvriothvroidism, OCMP	+ve	+ve		HCQ
8.	20	F	S E/10 Hypothyroidism	+ve	184		HCQ, Omnacortil,Azee
9.	29	F	SLE, Knee pain, Oral recurrent ulceration	+ve	+ve	+ve	HCQ
10.	18	F	SLE luous neohritis, lRTI, Anemia	+ve	+ve		HCQ, Azee
11.	20	F	SLE with lupus nephritis, UTI	+ve	+ve		HCQ, Omnacortil
12.	51	F	flA SL£ painin lov1er limb grom i:roin to knu Eleetrielikf sensation	+ve	+ve		HCQ
13.	18	F	SLE, Menorrhagia, Anemia, Facial edema Hypoalbuminea, Hvnothvroidism	+ve	+ve	+ve	HCQ, Cefixime
14.	32	F	S E	+ve	+ve		HCQ, Omnacortil
15.	28	F	S E	+ve	+ve		HCQ
16.	18	F	S E Heoatitis Hemolvricanemia	+ve	+ve		HCQ
17.	19	F	SLE, Lupus, Pneumonia, Koch's	+ve	+ve		HCQ
18.	28	M	SLE with Lupus nephritis (Stage 4 & 5) altered synovium & Seizure	+ve	+ve		HCQ
19.	55	F	SLE MCTD Anemia	+ve	*-ve*	+ve	HCQ
20.	30	F	SLE, Severe anemia with Jaundice	+ve	+ve		HCQ
21.	15	M	SLE, Small vesselvasculitis with diffuse alveolar hemorrhage Dermatomyositis	+ve	*-ve*		HCQ
22.	26	F	SLE with upus nephritis oericardial effusion	+ve	*-ve*	+ve	ZYQ, Omnacortil, MMF,
23.	32	F	SLE, APS, UTI	+ve	+ve	+ve	HCQ, Omnacortil
24.	27	F	SLE, Thrombocytopenia	+ve	+ve	+ve	Omnacortil
25.	23	F	SLE v1ithlupus nephritis, Acute Cutaneous lupus, cellulitis, emohvsemii	+ve	+ve		HCQ, LNZ
26.	24	F	SLE/CNS lupus	+ve	+ve		ZYQ, MMF, Omnacortil
27.	28	F	SLE with lupus nephritis	+ve	+ve		ZYQ, MMF, Omnaco rtil
28.	33	F	SLE	•ve	*-ve*	+ve	ZYQ Omnaeortil
29.	22	F	SLE, MCTD, Dermatomyositis	+ve	+ve	+ve	Omnacortil
30.	45	F	SLE, MCTD, PSS, hypothyroidism	+ve	+ve	+ve	HCQ, Omnacortil
31.	22	F	SLE AOCD	+ve	+ve		HCQ
32.	24	F	SLE, Seizure	+ve	+ve		HCQ, Omnacortil, MMF,
33.	20	F	SLE, MCTD, PSS	+ve	+ve	+ve	ZYQ, PAH
34.	21	F	SLE with lupus nephritis with Anemia	+ve	+ve		HCQ, Omnacortil
35.	49	F	SLE	+ve		+ve	HCQ
36.	15	F	SLE	+ve	+ve		HCQ, Omnacortil
37.	45	F	SLE	+ve	+ve		HCQ, Omnacortil
38.	35	F	SLE,CNS lupus, LRTI,PAH,AKI, CNS Vascul itis	+ve		+ve	MMF
39.	40	F	SLE, COPO, PSS, Grade 1PAH	+ve	+ve		HCQ
40.	23	F	SLE with lupus nephritis. acute psychosis	+ve	+ve	+ve	HCQ, Omnacortil, Nicardia
41.	25	F	SLE with luous neohritis	+ve	+ve		HCQ, Omnacortil
42.	36	F	SLE with lupus nephritis, AKI, AOCD	+ve	+ve		HCQ, Omnacortil

#### 3.1.1 DW and IW treatment reduced, cytotoxicity and NET associated markers in SLE patients’ neutrophils *ex vivo*

**Figure 1:**
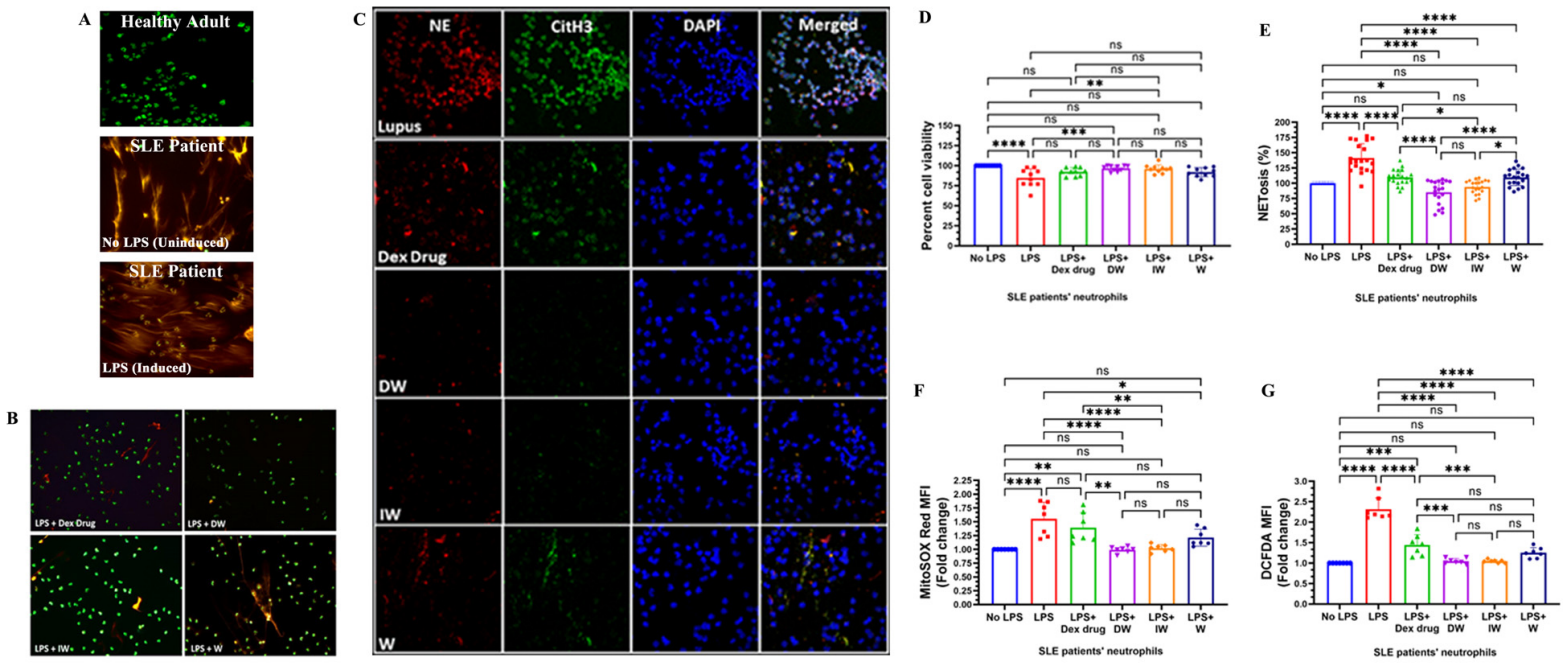
Immunomodulatory effect of DW and IW on NET formation, neutrophil viability and ROS generation in LPS induced SLE patients’ neutrophils *ex vivo*. The image reveals **(A)** NET formation in healthy adults and SLE patients’ neutrophils **(B)** decreased NET formation and **(C)** low expression of NET associated markers in DW and IW treated cells. NET formation was detected by confocal microscopy using two binding dyes; Sytox orange (red) and syto13 (green). Netting neutrophils were confirmed (as NE/CitH3/DNA overlap) by immunofluorescence staining with anti-NE (red), CitH3 (green) and DNA (blue). (At 40X, Scale Bar -100 um). The data in this figure are from 3 independent experiments. DW and IW treatments restored the compromised **(D)** cell viability (n=9) and **(E)** NETosis (n=20) incurred by LPS induction. DW and IW treatment suppress **(F)** mitochondrial as well as **(G)** cytoplasmic ROS generation in LPS induced SLE patients’ neutrophils. The line graphs illustrate mean data from a sample OF 6 SLE patient. Error bars show mean ± SEM. p values indicate significant changes as follows: non-significant (ns) p > 0.05, *p < 0.05, **p < 0.01, ***p < 0.001 and ****p < 0.0001; One-way ANOVA.

Staining with extracellular (Sytox orange) and intracellular (syto13) DNA binding dyes revealed an increased level of NET formation in neutrophils isolated from SLE patients, in both LPS-induced and uninduced conditions, compared to healthy adults (**Figure 1A**). The confocal microscopy images revealed that both DW and IW treatments were able to decrease the extracellular DNA release more effectively compared to Dexamethasone drug and W treatment (**Figure 1B**). Distinctive reduction in these NET-associated proteins (NE, CitH3) alongside extracellular DNA was evident in both DW and IW treated neutrophils (**Figure 1C**).

LPS induction resulted in a substantial decrease in cell viability compared to the uninduced condition Notably, both DW and IW treatments effectively restored the compromised cell viability incurred by LPS induction (**Figure 1D**). To validate our qualitative confocal microscopy based findings, we assessed the extracellular DNA release quantitatively, in the culture supernatants of cells with different treatments, and observed that the confocal images corroborated with our quantitative data, highlighting that DW and IW treatments exhibited a statistically significant reduction in NETosis compared to other conditions (**Figure 1E**).

ROS generation is crucial in driving the intricate pathophysiology of SLE, particularly in association with NET formation. The structural integrity of NETs, encompassing components such as histones, granular enzymes, and ROS, plays a pivotal role in mediating tissue damage in SLE patients (13). Our observations on ROS assessment in LPS-induced neutrophils treated with Dexamethasone drug, DW, IW and W revealed a significant reduction in both mitochondrial (**Figure 1F**) and cytoplasmic (**Figure 1G**) ROS generation with DW and IW treatments. These findings substantiate the potential of DW and IW in mitigating the oxidative stress associated with NETosis in SLE.

#### 3.1.2 DW and IW treatment reduced extracellular DNA release, ROS, and cytotoxicity in SLE patients’ neutrophils

**Figure 2:**
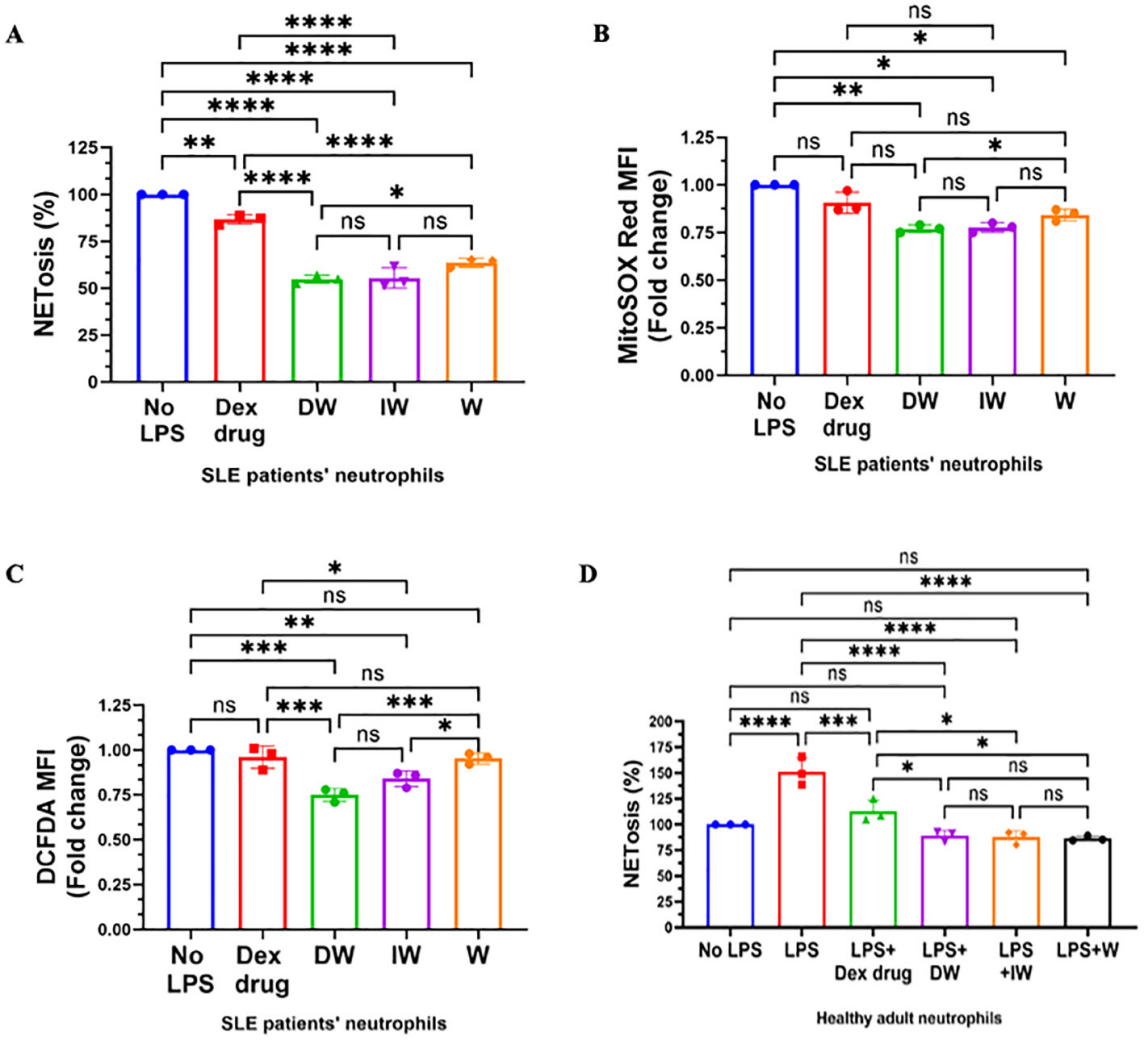
Immunomodulatory effect of DW and IW on NETosis and ROS generation in non-induced SLE patients’ neutrophils and healthy adult. DW and IW treatment suppress **(A)** extracellular DNA release **(B)** mitochondrial as well as **(C)** cytoplasmic ROS generation in uninduced SLE patients’ (n=3) neutrophils and **(D)** LPS induced NETosis in healthy adults (n=3). The line graphs illustrate mean data from a sample of 3 SLE patient and healthy adult. Error bars show mean ± SEM. p values indicate significant changes as follows: non-significant (ns) p > 0.05, *p < 0.05, **p < 0.01, ***p < 0.001 and ****p < 0.0001; One-way ANOVA.

Peripheral blood low-density granulocytes from SLE patients demonstrate a significantly heightened propensity for NET formation, in contrast to both healthy control neutrophils and autologous lupus neutrophils. Our findings indicated that, both DW and IW treatments efficiently attenuated NETosis (**Figure 2A**) and demonstrated a substantial reduction in the generation of both mitochondrial (**Figure 2B**) and cytoplasmic ROS (**Figure 2C**) both uninduced or LPS-induced neutrophils emphasizing the modulatory effectiveness of DW and IW treatments.

We also examined the impact of DW, IW, along with Dexamethasone drug and W treatments on extracellular DNA release in LPS-induced neutrophils isolated from healthy adults (n=3). It is worth noting that LPS induction resulted in a substantial increase in NETosis compared to the uninduced state. Interestingly, both DW and IW treatments demonstrated a statistically significant decrease in NETosis compared to other conditions *ex vivo* in healthy adult sample (**Figure 2D**).

#### 3.1.3 DW and IW treatment significantly reduced NET associated markers in SLE patients’ neutrophils stimulated with RNP-IC^+^ serum

Presence of RNP ICs are characteristic to SLE and are associated with interferon gene signature. Upon stimulating neutrophils with RNP-ICs, an elevation in mitochondrial ROS levels, leading to mitochondrial depolarization and the subsequent release of NETs enriched in oxidized mitochondrial DNA has been known (14). This motivated us to investigate the impact of DW and IW treatment on various parameters of RNP-IC^+^ serum (10 µl) induced NETosis. For experiments aiming to induce NETosis and ROS generation, we selected the lowest effective volume of RNP IC-positive serum (10 µl) (**Supplementary Figures 2A– D**).

**Figure 3:**
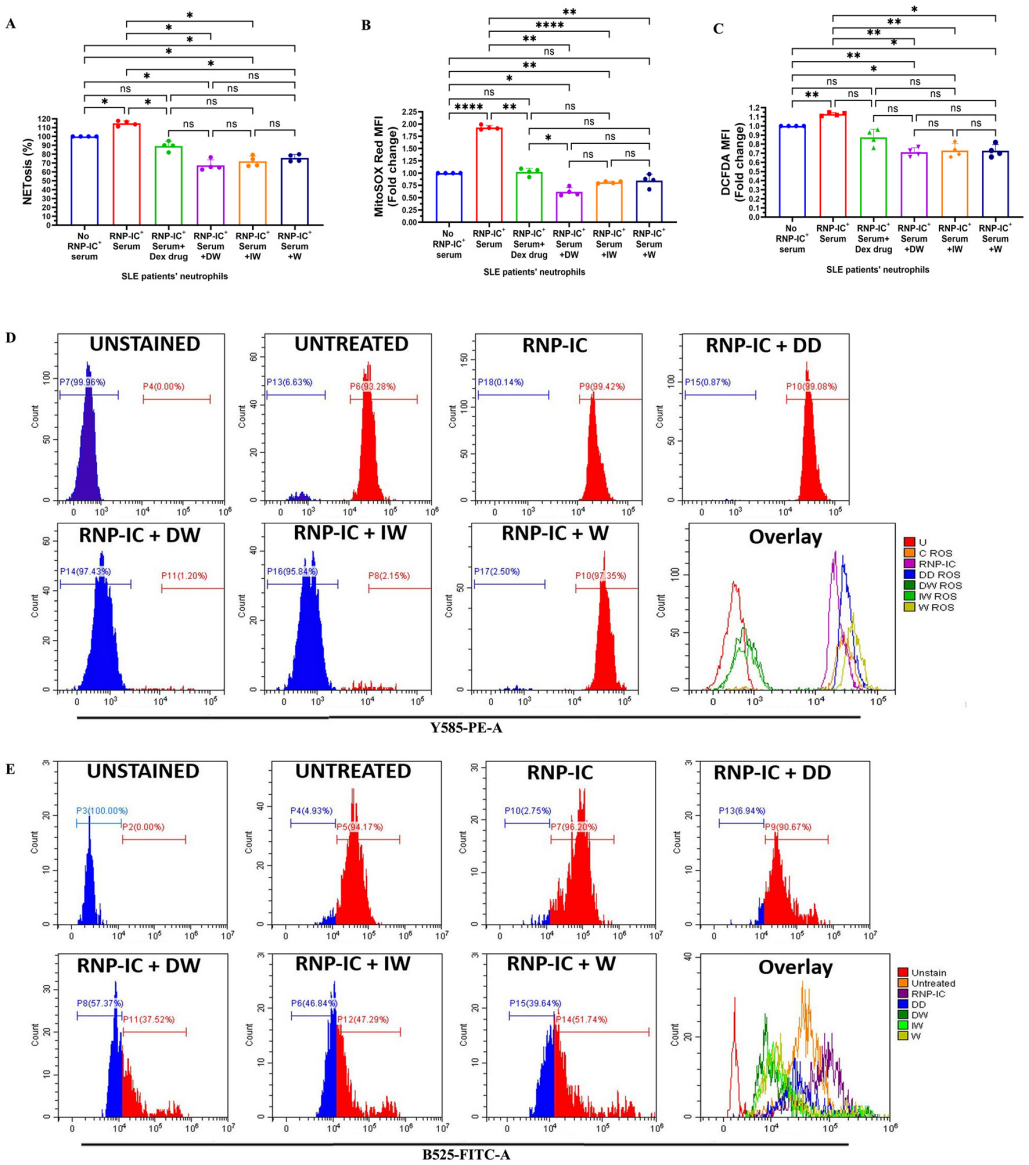
Immunomodulatory effect of DW and IW on NETosis and ROS generation in RNP-IC induced SLE patients’ neutrophils. DW and IW treatment suppress **(A)** extracellular DNA release and **(B)** mitochondrial as well as **(C)** cytoplasmic ROS generation in RNP-IC induced SLE patients’ neutrophils. **(D)** Mitochondrial and **(E)** cytoplasmic ROS was also determined by flow cytometry. The line graphs illustrate mean data and histogram represent individual data from a sample of 3 SLE patient. Error bars show mean ± SEM. p values indicate significant changes as follows: non-significant (ns) p > 0.05, *p < 0.05, **p < 0.01 and ****p < 0.0001; One-way ANOVA.

Both DW and IW treatments demonstrated a significant reduction in NETosis, as evidenced by a substantial decrease in extracellular DNA release (**Figure 3A**) and a pronounced decrease in mitochondrial as well as cytoplasmic ROS generation (**Figures 3B, C**), in neutrophils activated by RNP-IC positive serum. Flow cytometric analysis for cytoplasmic and mitochondrial ROS generation further validated the suppressive impact of DW and IW (**Figures 3D, E**).

#### 3.1.4 DW/IW and standard drug HCQ combination reduces NET related changes more effectively than HCQ alone

**Figure 4:**
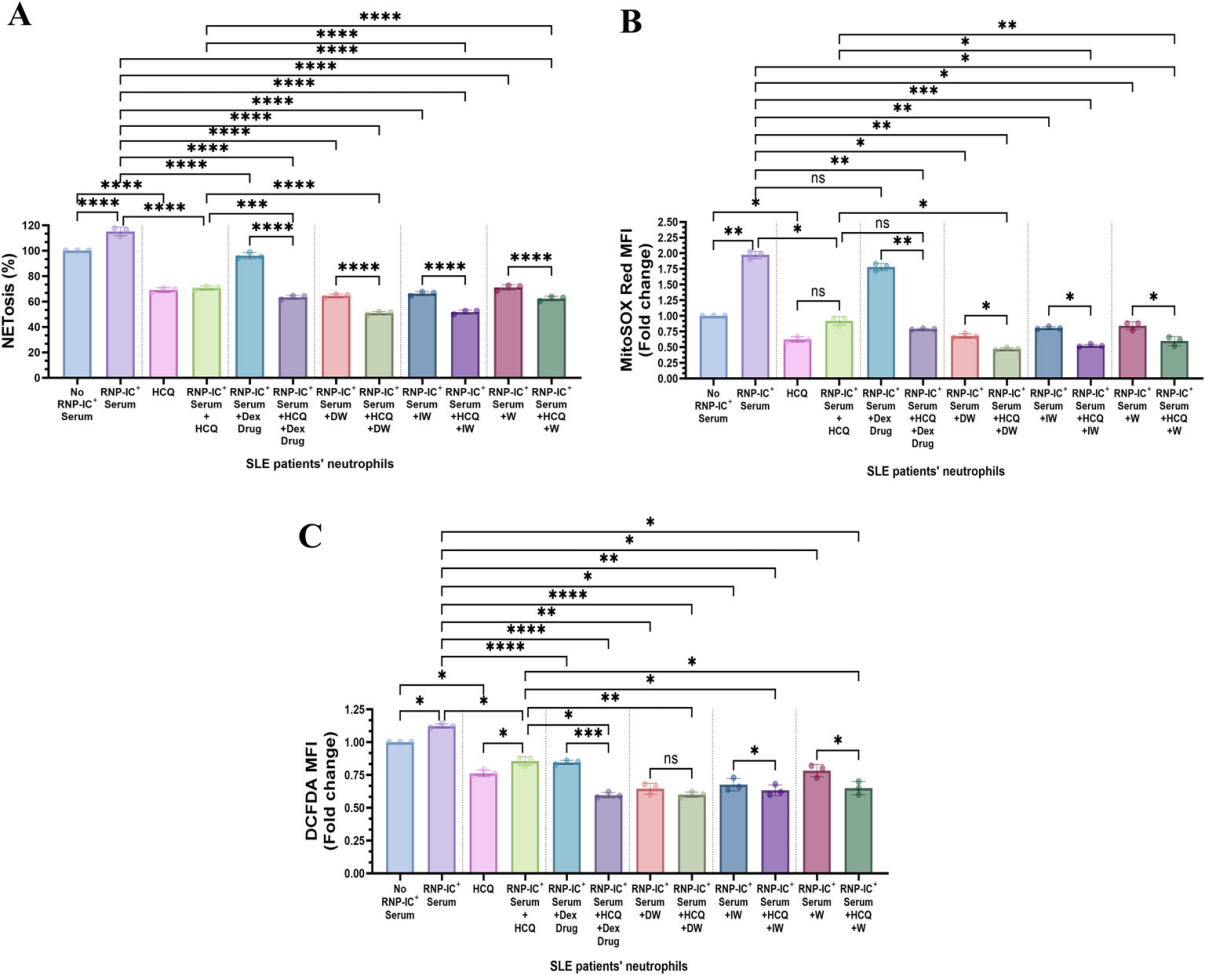
Immunomodulatory effect of DW/IW and HCQ combination on NETosis and ROS generation in RNP-IC positive serum induced SLE patients’ neutrophils. DW/IW and HCQ treatment suppress **(A)** extracellular DNA release and **(B)** mitochondrial as well as **(C)** cytoplasmic ROS generation in RNP-IC induced SLE patients’ neutrophils. The line graphs illustrate mean data from a sample of 3 SLE patient. Error bars show mean ± SEM. p values indicate significant changes as follows: non-significant (ns) p > 0.05, *p < 0.05, **p < 0.01, ***p < 0.001 and ****p < 0.0001; One-way ANOVA.

HCQ and corticosteroids, integral components of SLE drug therapy, have demonstrated the capacity to decrease NET formation *ex vivo*. In our study, we assessed the impact of HCQ alone and in combination with DW and IW on NETosis and ROS generation in RNP-IC^+^ serum induced neutrophils from SLE patients. Interestingly, DW and IW, both individually and in combination (DW+HCQ and IW+HCQ), exhibited a significantly higher reduction in NETosis compared to HCQ alone (**Figure 4A**). Importantly, the combination therapy demonstrated similar outcomes to DW and IW used alone, indicating that DW and IW contribute dominantly towards the reduction in NET formation. In addition, combination therapy involving HCQ with DW or IW showcased a pronounced reduction in both mitochondrial and cytoplasmic ROS levels compared to HCQ alone (**Figures 4B, C**).

#### 3.1.5 DW and IW immunomodulates NETosis through IDO and PGE2

**Figure 5:**
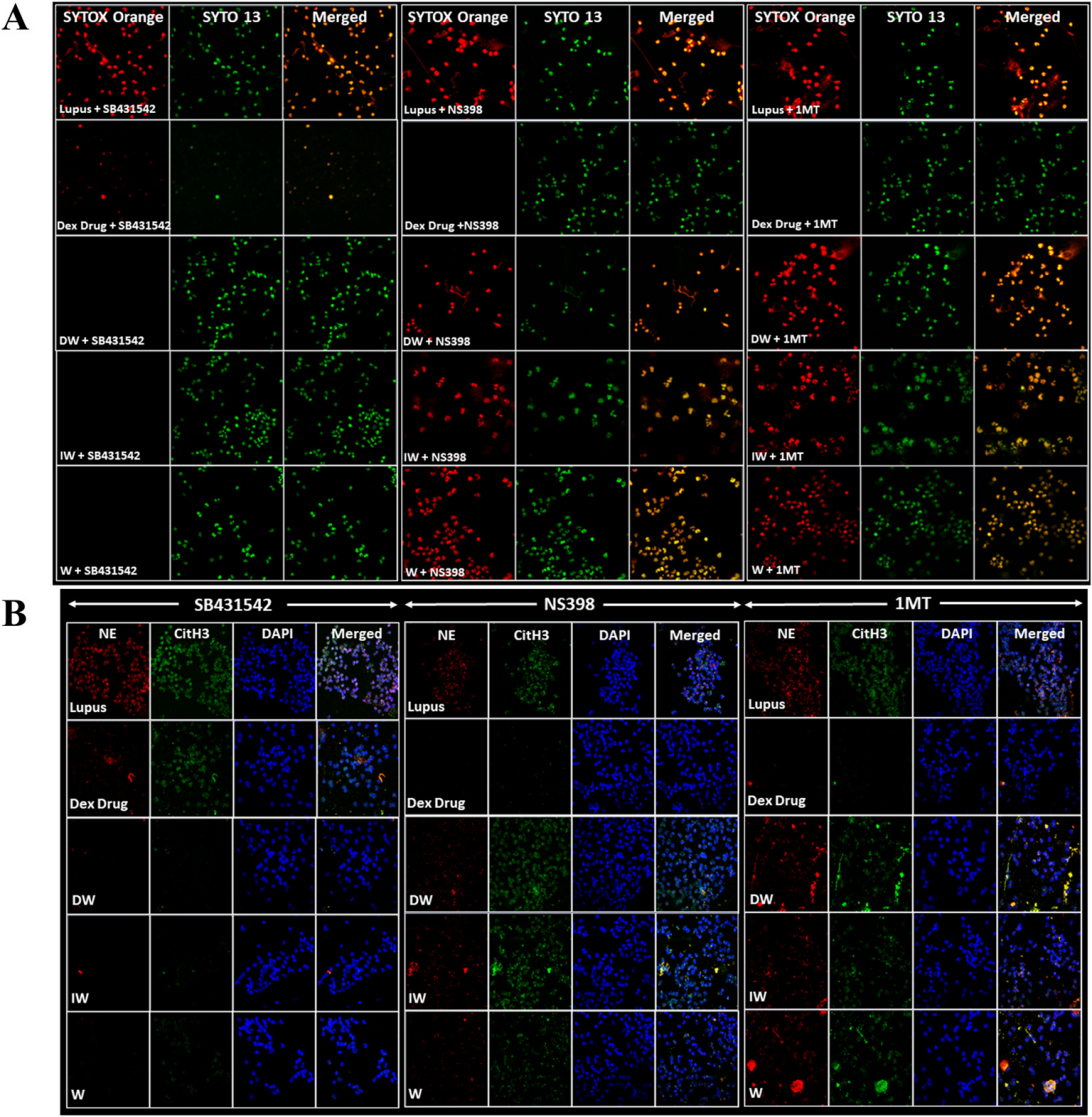
DW and IW immunomodulates NETosis and ROS generation through IDO and PGE2. The image reveals **(A)** NET formation **(B)** NET associated markers in SLE patients’ neutrophils upon inhibition by using specific inhibitors for TGF-β (SB-431542), PGE2 (NS398) and IDO (1MT). NET formation was detected by confocal microscopy using two binding dyes; Sytox orange (red) and syto13 (green). Netting neutrophils were confirmed (as NE/CitH3/DNA overlap) by immunofluorescence staining with anti-NE (red), CitH3 (green) and DNA (blue). (At 63X objective, Scale Bar -100 um). The data in this figure are from 3 independent experiments.

Major immunomodulatory factors secreted by WJ-MSCs include PGE2, IDO and TGF-β. To investigate the role of these molecules in the suppression of NETosis by DW and IW, inhibition studies were conducted using specific inhibitors for TGF-β (SB-431542 hydrate), PGE2 (NS398) and IDO (1MT). The impact of DW and IW treatments on NET formation and ROS generation in neutrophils from patients with SLE was examined in the presence of SB431542 + NS398, NS398 + 1MT, or 1MT + SB431542 hydrate. Results suggested that inhibiting TGF-β did not produce any significant change in NET formation as compared to DW and IW treatment. This suggests that presence of PGE2 and IDO and not TGF-β, facilitated the immunomodulatory action of DW and IW in mediating the suppression of NET (**Figures 5A, B**). Conversely, inhibition of IDO or PGE2 prior to DW or IW treatment did not result in reduction of NETosis as was seen with DW or IW treatment in absence of these inhibitors (**Figures 1B, C**). These findings impresses upon the pivotal roles played by IDO and PGE2 in the DW and IW-mediated suppression of NETosis.

##### 3.1.5.1 Double Inhibition study

**Figure 6:**
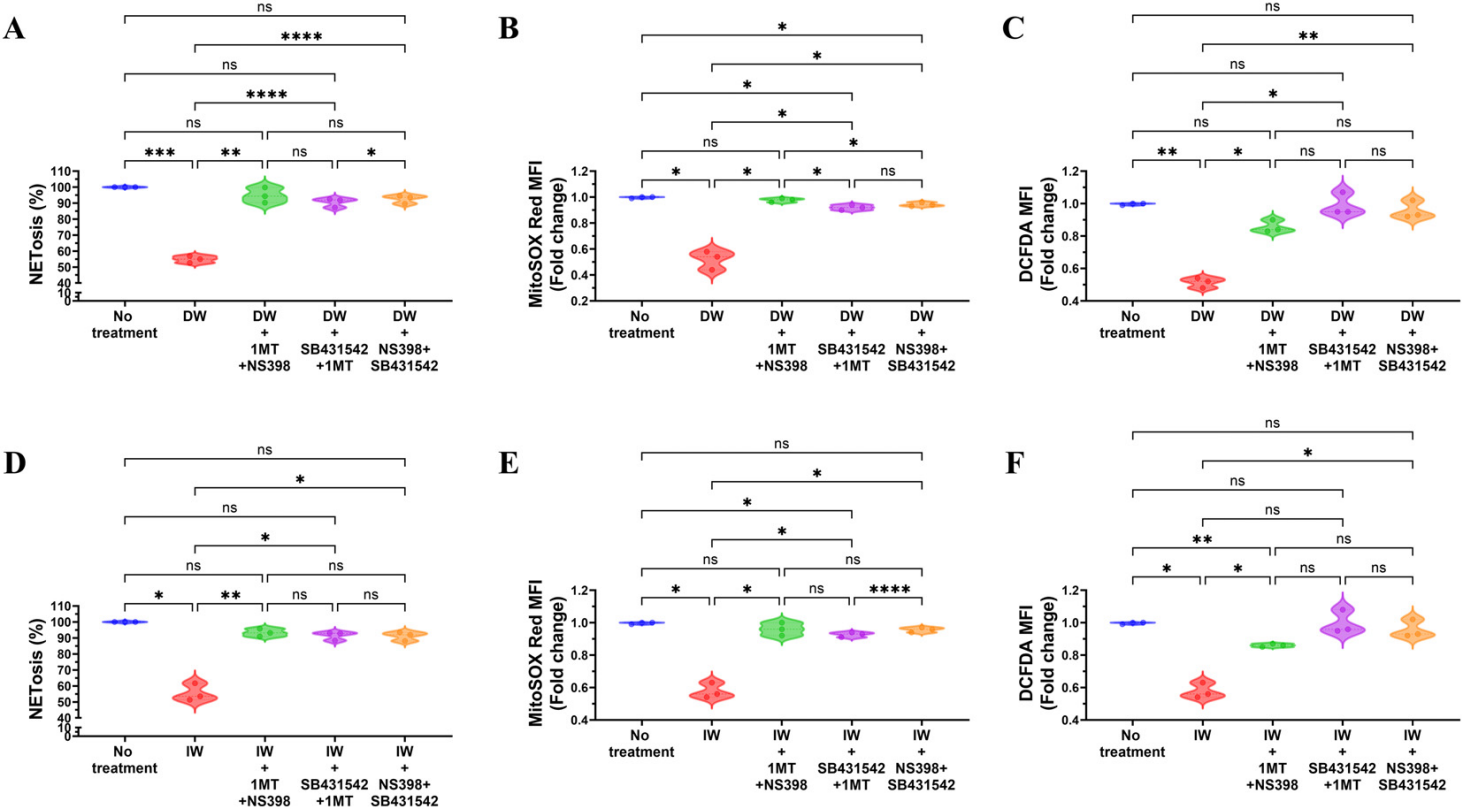
Role of TGF-β and IDO, TGF-β and PGE2, IDO and PGE2 together in DW and IW mediated suppression of NETosis and ROS generation. The data reveals changes in NETosis, as well as mitochondrial and cytoplasmic ROS generation upon inhibition of **(A)** TGF-β and IDO, TGF-β and PGE2 and IDO and PGE2 in DW **(A–C)** and IW treated neutrophils **(D–F)**. The violin plot illustrate mean data from a sample of 3 SLE patient. Error bars show mean ± SEM. p values indicate significant changes as follows: non-significant (ns) p > 0.05, *p < 0.05, **p < 0.01, ***p < 0.001 and ****p < 0.0001; One-way ANOVA.

The double inhibition studies indicated that presence of TGF-β along with either IDO or PGE2, a reduction in NETosis and ROS was observed, enhancing our understanding of the immunomodulatory mechanisms orchestrated by DW and IW through IDO and PGE2 in the regulation of NETosis and ROS. Results demonstrated that inhibiting both TGF-β and IDO, TGF-β and PGE2, IDO and PGE2 in combination, led to increased NETosis and ROS production in case of DW (**Figures 6A–C**) and IW (**Figures 6D–F**) treatment compared to uninhibited counterparts, as was previously observed while inhibiting one molecule at a time (either of IDO or PGE2). This implies that the presence of IDO and PGE2 in DW and IW significantly contributed to the observed suppression of NETosis and ROS.

### 3.2 *In vivo* studies in PIL model

#### 3.2.1 Development of PIL model

The pristane-injected group demonstrated a significant increase in autoantibodies (both anti-dsDNA and anti-ENA) compared to the pre-treatment levels in a span of 35 days (**Supplementary Figures 3A, B**). Urinary protein was quantified from urine samples of individual mice in metabolic cages on 35^th^ day of treatment. Urinary protein excretion in pristane-injected mice on 35^th^ days was significantly higher than that of the pre-treatment levels (**Supplementary Figure 3C**).

To assess NET formation and ROS generation in the pristane injected group of mice quantitative assessment was done which revealed an elevated level of extracellular DNA (**Supplementary Figure 3D**) and ROS (**Supplementary Figures 3E, F**) in neutrophils isolated from PIL mice compared to neutrophils from healthy control mice was significantly higher than that of the pre-treatment levels and healthy mice (control group).

Over a period of two months, 42 female BALB/c mice were raised, with 35 mice received intraperitoneal pristane over the next month to induce lupus-like autoimmunity (15). Unimmunized mice (n=7) served as controls. Following the development of autoantibodies and SLE like symptoms and pathology, the pristane-induced mice were divided into four groups (7 mice per group): i) lupus (treated with normal saline), ii) Dexamethasone drug (received Dexamethasone drug treatment), iii) DW (received Dexamethasone-primed CM treatment), iv) IW (received IFN- γ primed CM treatment) and v) W (received CM treatment).

#### 3.2.2 DW and IW administration in lupus mice reduced formation of NET and associated proteins in neutrophils

**Figure 7:**
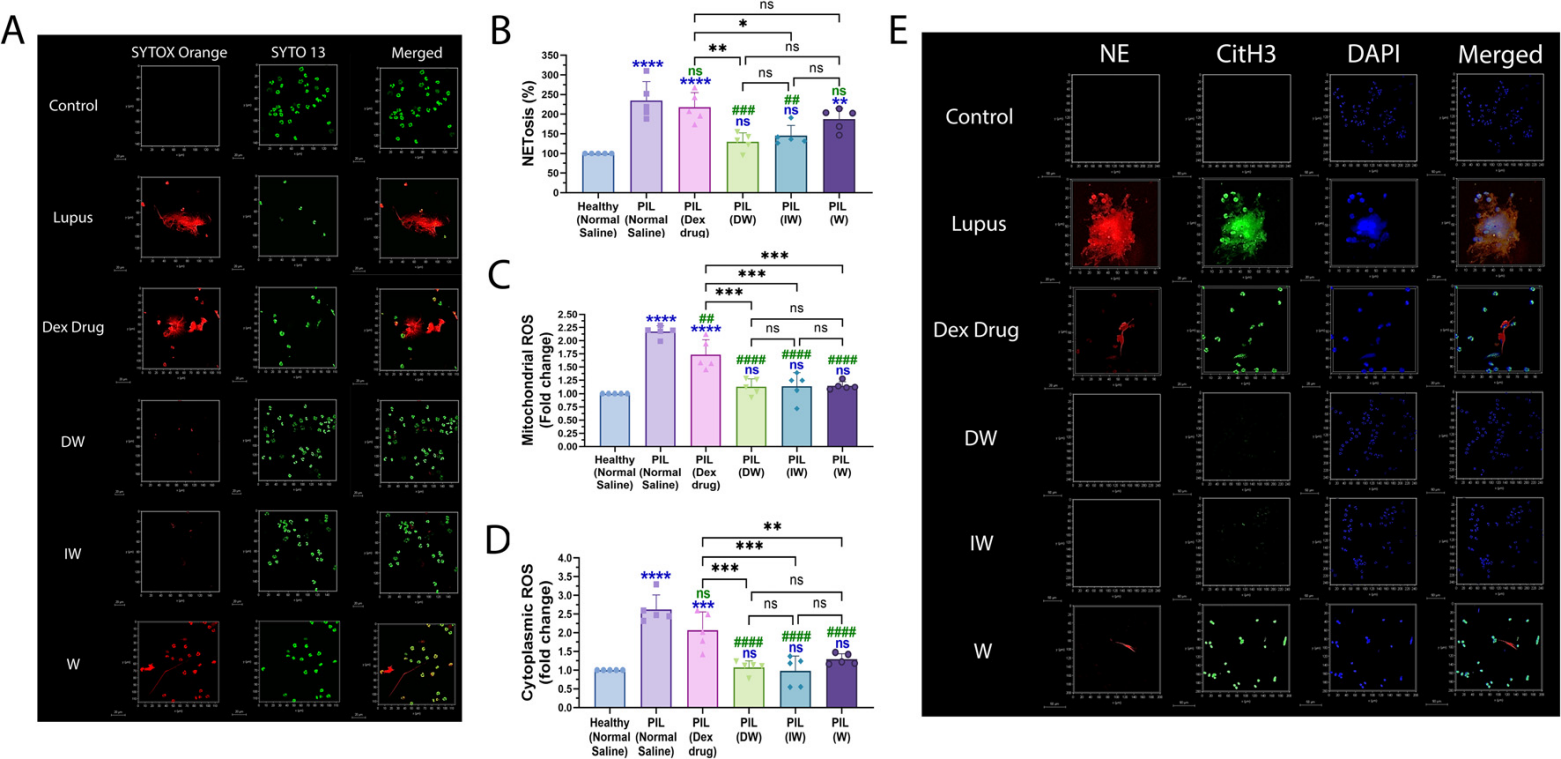
Immunomodulatory effect of DW and IW on NET formation and ROS generation in PIL mice. **(A)** The image reveals decreased NET formation in DW and IW treated PIL mice. NET formation was detected by confocal microscopy using two binding dyes; Sytox orange (red) and syto13 (green). DW and IW treatment suppress **(B)** extracellular DNA release **(C)** mitochondrial as well as **(D)** cytoplasmic ROS generation in PIL mice. The line graphs illustrate mean data from 5 mice. **(E)** The image reveals low expression of NET associated markers (CitH3 and NE) in DW and IW treated PIL mice. Netting neutrophils were confirmed (as NE/CitH3/DNA overlap) by immunofluorescence staining with anti-NE (red), CitH3 (green) and DNA (blue). (At 63X, Scale Bar - 20 µm). The data in this figure are from 3 independent experiments. Error bars show mean ± SEM. p values indicate significant changes as follows: non-significant (ns) p > 0.05, *p < 0.05, **p < 0.01, ***p < 0.001 and ****p < 0.0001; One-way ANOVA. ##P < 0.01, ###P < 0.001, ####P < 0.0001 and ns (non-significant). * - compared to Healthy (Normal Saline) and # - compared to PIL (Normal Saline).

To validate the *ex vivo* findings pertaining to the immunomodulatory effects of DW and IW in suppressing NET formation and ROS generation as observed in neutrophils from patients with SLE, we employed PIL murine model. Confocal microscopy imaging of NET formation using extracellular (Sytox orange) and intracellular (syto 13) DNA-binding dyes along with quantitative assessment revealed an elevated level of extracellular DNA (**Figures 7A, B**) and ROS (**Figures 7C, D**) in neutrophils isolated from PIL mice compared to neutrophils from healthy control mice with DW and IW administration. Furthermore, we also examined the expression of NET-associated granular proteins NE and CitH3, in neutrophils and noticed reduced levels of NET-associated proteins NE, CitH3 in neutrophils of mice treated with DW and IW (**Figure 7E**).

#### 3.2.3 Both DW and IW efficiently reduced extracellular DNA release in liver, kidneys and heart of PIL mice

The intricate relationship between ROS production, NET formation, and associated chromatin decondensation, particularly in lupus, has been extensively studied (5). In this study, we present the assessment of two contrast agent Sytox Orange and MPO for *in vivo* application.

**Figure 8:**
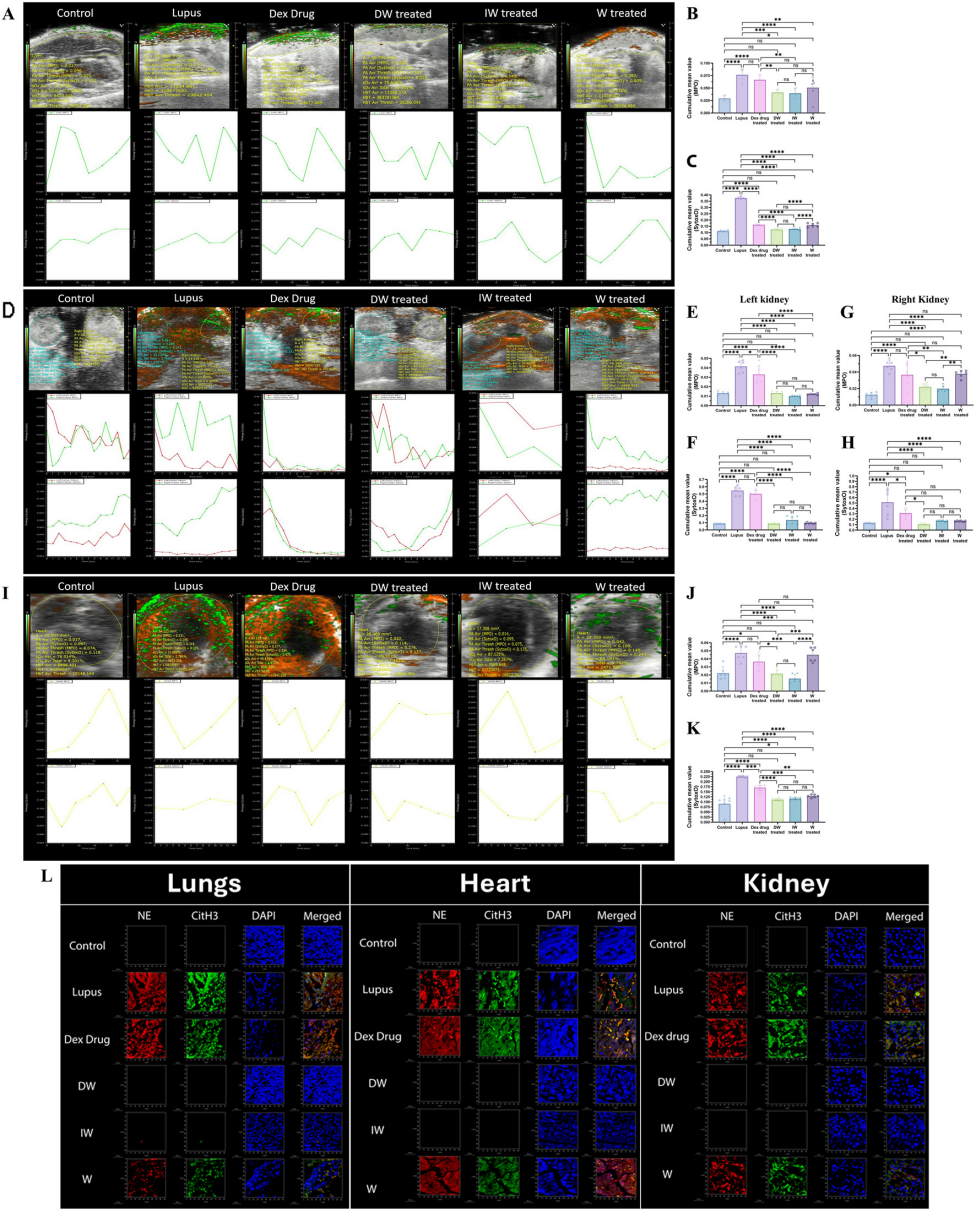
Immunomodulatory effect of DW and IW on extracellular DNA release and MPO in different organs of PIL mice. **(A)** Both DW and IW efficiently reduced extracellular DNA release and MPO in **(A–C)** liver, **(D–H)** kidneys and **(I–K)** heart of PIL mice *in vivo* as observed in PAI system. **(B, C)** Data are presented as mean ± standard deviation from n=6 mice/group. Error bars show mean ± SEM. p values indicate significant changes as follows: non-significant (ns) p > 0.05, *p < 0.05, **p < 0.01, ***p < 0.001 and ****p < 0.0001; One-way ANOVA. The color green denotes MPO and red denotes Sytox orange. **(L)** DW and IW reduced NET associated markers, NE and CitH3 in lungs, heart and kidney of PIL mice. The image reveals low expression of NET associated markers (CitH3 and NE) in different organs upon DW and IW treatment in PIL mice. NET formation was detected by confocal microscopy. Netting neutrophils were confirmed (as NE/CitH3/DNA overlap) by immunofluorescence staining with anti-NE (red), CitH3 (green) and DNA (blue). (At 63X, Scale Bar - 1000 µm). The data in this figure are from 3 independent experiments.

The tissue-specific accumulation of MPO and Sytox Orange was systematically assessed in the liver, kidneys, and heart of mice across various treatment groups, lupus, Dexamethasone drug, DW, IW, and W treated mice along with healthy control mice. Imaging was performed using the PA Vevo^®^ LAZR system (FUJIFILM VisualSonics) at 680 nm, 30 min post-injection. Our observations align with the trends presented in **Figure 7A**, revealing distinctive patterns of Sytox Orange and MPO accumulation in liver (**Figures 8A–C**), kidneys (**Figures 8D–H**), heart (**Figures 8I–K**) of lupus and treated mice across all examined organs. Notably, lupus mice exhibited a significant and pronounced increase in both Sytox Orange and MPO compared to the healthy control group. Lupus mice displayed heightened levels of Sytox Orange and MPO, indicating an enhanced presence of extracellular DNA and NET-associated proteins. This substantial augmentation in lupus mice was consistently observed across all organs, corroborating the systemic nature of the disease. Conversely, mice treated with DW and IW exhibited a significant reduction in both Sytox Orange and MPO accumulation. This reduction was evident through diminished staining for extracellular DNA (Sytox Orange) and NET-associated proteins (MPO), presenting a notable contrast to the heightened levels observed in lupus, Dexamethasone drug and W treated counterparts.

#### 3.2.4 DW and IW treated lupus mice showed reduced expression of NET-associated markers in lungs, heart and kidneys of PIL mice

Following a 42-day treatment period, euthanasia was performed using carbon dioxide (20-25% of chamber volume per minute), mice were sacrificed and their kidneys, liver, and heart were harvested for subsequent staining for NE and CitH3. For measuring the tissue-specific accumulation of MPO in the liver, kidneys, and heart of mice across various treatment groups, confocal imaging was done. In congruence with the observations in **Figure 7E**, a pronounced reduction in NET-associated proteins NE, CitH3 and MPO was observed in mice receiving DW or IW treatment (**Figure 8L**).

## 4 Discussion

NETs are immunogenic structures that are recognized by autoantibodies in SLE patients, targeting dsDNA, histones, or nucleosomes. Both excessive formation and impaired degradation of NETs are implicated in SLE pathophysiology (16). SLE patients with defective NET degradation exhibit elevated anti-NET and anti-dsDNA autoantibodies, with a higher lupus nephritis incidence (Hakkim et al., 2010). Arresting the NET process has emerged as one of the potential line of therapeutic strategy for improving the disease outcomes. In recent years, the exploration of priming approaches has aimed to enhance the immunomodulatory effectiveness of MSCs (17). A notable strategy involves preconditioning MSCs with various preconditioning agents. One such preconditioning agent is IFN-γ, which under hypoxic conditions enhances the immunosuppressive properties of MSCs. With IFN-γ priming (17) MSCs exhibit increased expression of class II HLA molecules. IFN-γ appears to function as a master regulator upstream of various inflammatory and regulatory pathways, contributing to a self-regulatory process crucial for immune system homeostasis. This self-limiting capability prevents most inflammatory processes from progressing into pathological conditions.

Earlier our collaborative group showed that Dexamethasone also holds potential of priming MSCs towards better immunomodulation (18). Dexamethasone is a widely recognized anti-inflammatory glucocorticoid and has demonstrated anti-inflammatory properties by modulating immune responses and inhibiting various pro-inflammatory pathways (19) and remarkable effectiveness in suppressing the formation of NETs in *ex vivo* studies. The ability of Dexamethasone to mitigate NET formation holds significant implications for understanding and managing inflammatory disorders, particularly those characterized by dysregulated immune responses. Several studies have provided substantial evidence supporting the inhibitory effects of Dexamethasone on NETosis. Dexamethasone was shown to effectively suppress NET release in murine models of acute lung injury (20). Another study highlighted the inhibitory role of Dexamethasone in preventing NET formation, release of NET-associated DNA and key proteins induced by various stimuli, including phorbol 12-myristate 13-acetate (PMA) and LPS (21). Neutrophils from SLE patients treated with Dexamethasone exhibited a decreased capacity for NET formation, suggesting the potential therapeutic value of Dexamethasone in managing NET-associated complications in autoimmune diseases Garcia-Romo et al. (22). Our earlier studies in SLE employing Dexamethasone or IFN-γ preconditioned MSCs have yielded significant disease ameliorating changes in the inflammatory profiles including reduction in autoantibodies, Th17 cytokines, Th17 cells, double negative T cells, inflammatory neutrophils population, organ pathology, organ physiology, improvement in T and B regulatory cells, and symptoms of alopecia, seizures, and limb inflammation. Hence, we used Dexamethasone and IFN-γ preconditioned media to assess their immunomodulatory effect on the process of NETosis in SLE.

Furthermore, DW and IW treatments exhibited a pronounced decrease in the expression of NET-associated granular proteins NE and CitH3, supporting their efficacy in mitigating NET formation in SLE. Interestingly, DW and IW not only suppressed NETosis but also exerted a protective effect on neutrophil viability following LPS induction. These findings were corroborated by confocal microscopy and quantitative assessments, reinforcing the potency of DW and IW in mitigating NETosis in SLE. The observed reduction in both mitochondrial and cytoplasmic ROS generation with DW and IW treatments is suggestive of their comprehensive impact on oxidative stress in SLE. In SLE, an upregulation of ROS levels in neutrophils, has been consistently associated with an augmented predisposition towards NETosis. This aberrant production of ROS not only acts as a trigger for initiating NET formation but also sustains the ongoing process. The heightened oxidative stress, originating from both cytoplasmic and mitochondrial sources, emerges as a robust inducer of NETosis within the SLE milieu. Furthermore, mitochondrial dysfunction in SLE can exacerbate the release of mitochondrial ROS, thereby exerting additional influence on the NETotic process (23). This intricate interplay between cytoplasmic and mitochondrial ROS dynamics underscores the multifaceted role of oxidative stress in fostering NETosis and elucidates its significance in the complex pathophysiology of SLE.

Concurrently, neutrophil oxidative metabolism analyses has revealed that SLE patients exhibited elevated basal-state oxidative activity compared to neutrophils from healthy controls (24). DW and IW treatments effectively attenuated NETosis and reduced ROS generation in both induced and uninduced neutrophils from SLE patients, demonstrating their consistent efficacy in modulating NET-related processes in SLE.

Presence of RNP ICs are characteristic to SLE and are associated with interferon gene signature (25). We studied the impact of DW and IW on NETosis induced by anti-RNP ICs, wherein significant reduction in NETosis and ROS generation induced by anti-RNP ICs could be observed with DW and IW treatments highlighting their potential in countering an autoimmune stimulus.

Major immunomodulatory factors secreted by WJ-MSCs include PGE2, IDO and TGF-β (26). IFN- γ has been shown to signal immunosuppression through IDO (27) and TGF-β (28). Studies including from our collaborative group on DW (18) have also indicated upregulated immunomodulatory molecules, PGE-2 and IDO in a dose-dependent manner. Presence of PGE2, IDO, and TGF-β in WJ-MSCs along with their involvement in the immunomodulation has also been suggested (29) Hence, to unravel the underlying mechanisms of DW and IW-mediated immunomodulation, we investigated the involvement of key immunomodulatory factors secreted by WJ-MSCs, namely PGE2, IDO, and TGF-β.

Inhibition studies revealed that while TGF-β inhibition did not significantly alter NETosis and ROS production, whereas individual inhibition of IDO and PGE2 resulted in a noteworthy reduction suggests that DW and IW mediated regulation on NETosis via IDO and PGE2. The collaborative effect of TGF-β with either IDO or PGE2 further highlighted their synergistic roles in mediating the immunomodulatory actions of DW and IW. HCQ and corticosteroids, integral components of SLE drug therapy, have demonstrated the capacity to decrease NET formation ex vivo (16). Importantly, the combination of DW and IW, either alone or in conjunction with HCQ, demonstrated superior efficacy in suppressing NETosis and ROS generation compared to HCQ alone in both induced and uninduced conditions. This suggests that DW and IW possess intrinsic immunomodulatory capabilities, could augment the therapeutic potential of standard SLE medications.

To validate our *ex vivo* findings, we extended our investigation to a PIL mouse model. Notably, DW and IW treatments in PIL mice model not only reduced NETosis and ROS generation but also ameliorated lupus severity, as evidenced by diminished staining for NET-associated proteins (NE, CitH3) expressions in the kidneys, liver, and heart. This *in vivo* validation together with our parallel findings on organs pathology and diseases manifestations strengthens the translational relevance of our study and highlights the potential of DW and IW as promising therapeutic agents in SLE.

In conclusion, our study elucidates the multifaceted impact of DW and IW on NETosis, ROS dynamics, and lupus severity on neutrophils from SLE patients and a murine lupus model. The robust and consistent efficacy of DW and IW, both *ex vivo* and *in vivo*, underscores their potential as innovative therapeutic strategies in the management of SLE. Further investigations into the specific mechanisms underlying their immunomodulatory actions are warranted, paving the way for the development of novel, targeted, cell free biological targeted and personalized approaches for SLE treatment.

## Data Availability

The original contributions presented in the study are included in the article/**Supplementary Material**. Further inquiries can be directed to the corresponding author.

## Supplementary material

Supplementary Figure 1 Phantom for Sytox orange dye and NETosis marker (MPO). Evaluation of a Sytox orange and MPO contrast agent for PAI in PIL mice: Phantom Validation and *In vivo* Performance.

Supplementary Figure 2 Evaluating the optimum concentration of RNP-ICs with varying serum concentrations (10, 20, and 40 µl) for inducing NETosis and ROS generation *ex vivo*. We compared various volumes of RNP IC-positive serum to determine the optimal volume not having **(A)** cytotoxicity for inducing significantly heightened **(B)** NETosis, **(B)** mitochondrial and **(C)** cytoplasmic ROS generation, The line graphs illustrate mean data from a sample of 3 SLE patient. Error bars show mean ± SEM. p values indicate significant changes as follows: non-significant (ns) p > 0.05, *p < 0.05, **p < 0.01, ***p < 0.001 and ****p < 0.0001; One-way ANOVA.

Supplementary Figure 3 Development of PIL mice model. Intraperitoneal injection with pristane significantly increased **(A)** anti-dsDNA, **(B)** anti-ENA, **(C)** urine protein **(D)** extracellular DNA **(E)** mitochondrial as well as **(F)** cytoplasmic ROS in a span of 35 days as compared to the pre-injected levels in mice (n=35). The bar graphs illustrate mean data from a sample of 35 mice. Error bars show mean ± SEM. p values indicate significant changes as follows: ****p < 0.0001; Student T-test.
