# Supplementary figures and images for: Dexamethasone and IFN-γ primed mesenchymal stem cells conditioned media immunomodulates aberrant NETosis in SLE via PGE2 and IDO
Source: Front Immunol. 2024 Oct 31;15:1461841. doi: 10.3389/fimmu.2024.1461841 (PMC11560778; doi:10.3389/fimmu.2024.1461841)

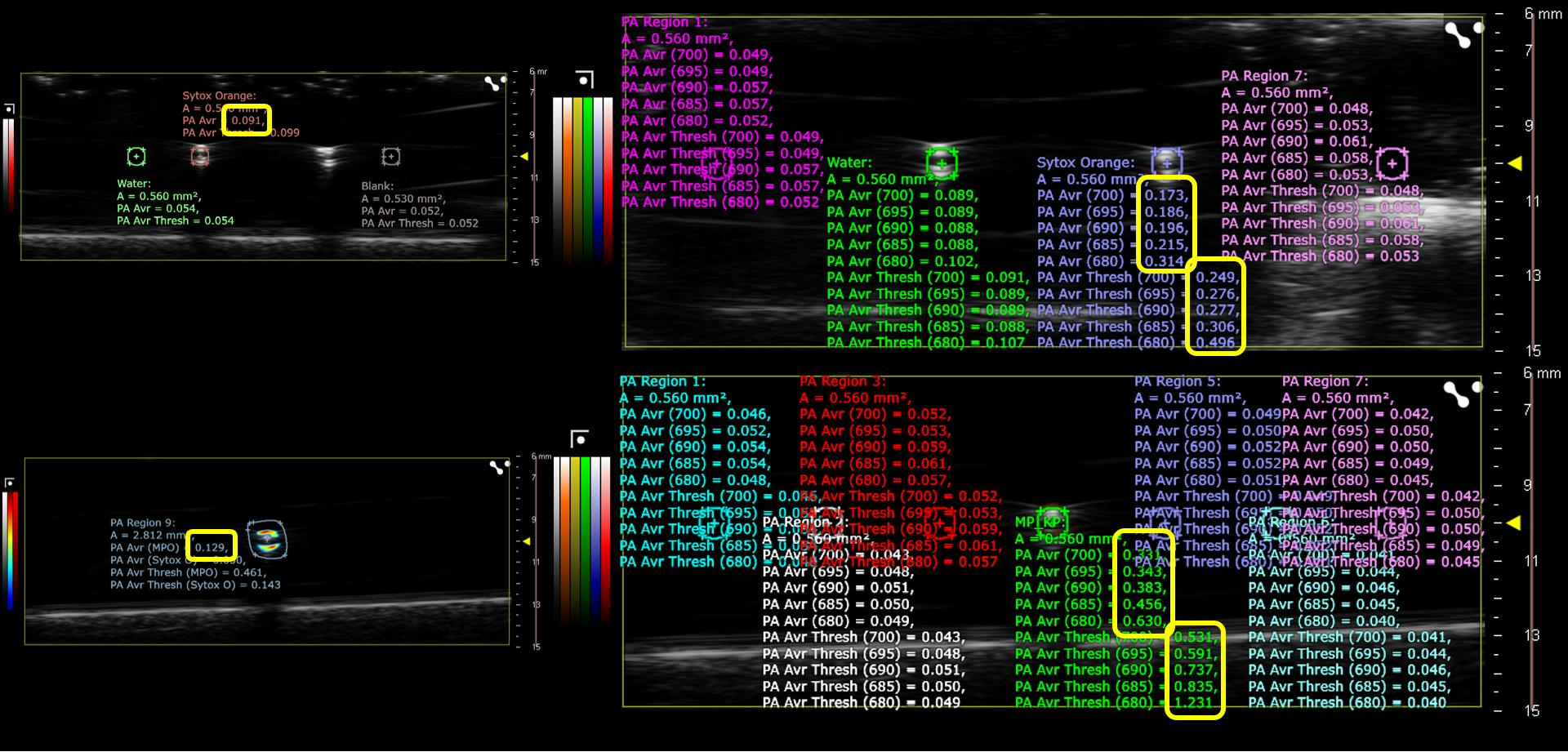

Supplement: Supplementary Figure 1 — Phantom for Sytox orange dye and NETosis marker (MPO). Evaluation of a Sytox orange and MPO contrast agent for PAI in PIL mice: Phantom Validation and In vivo Performance. [file Image1.tiff]

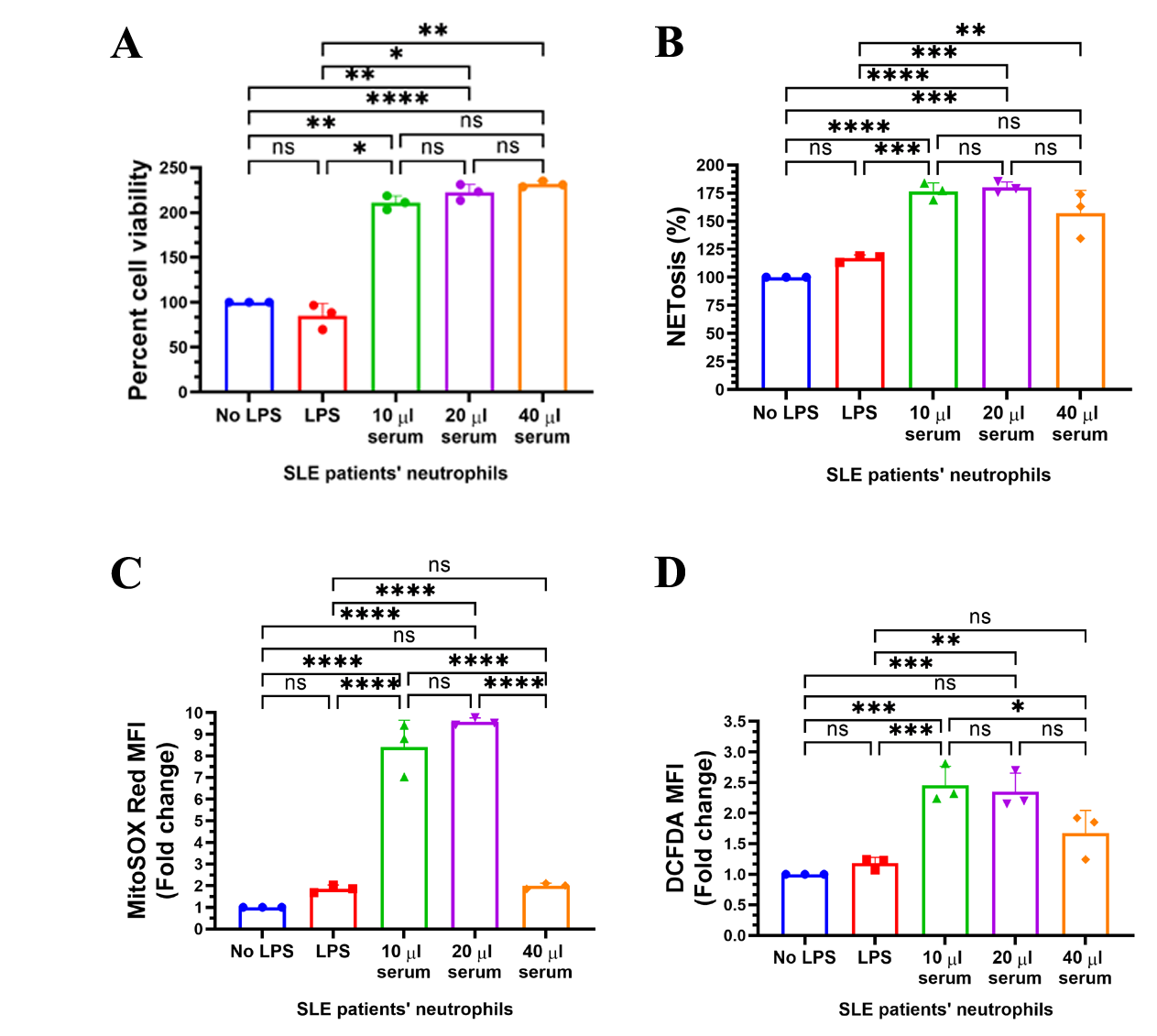

Supplement: Supplementary Figure 2 — Evaluating the optimum concentration of RNP-ICs with varying serum concentrations (10, 20, and 40 µl) for inducing NETosis and ROS generation ex vivo. We compared various volumes of RNP IC-positive serum to determine the optimal volume not having (A) cytotoxicity for inducing significantly heightened (B) NETosis, (B) mitochondrial and (C) cytoplasmic ROS generation, The line graphs illustrate mean data from a sample of 3 SLE patient. Error bars show mean ± SEM. p values indicate significant changes as follows: non-significant (ns) p > 0.05, *p < 0.05, **p < 0.01, ***p < 0.001 and ****p < 0.0001; One-way ANOVA. [file Image2.tif]

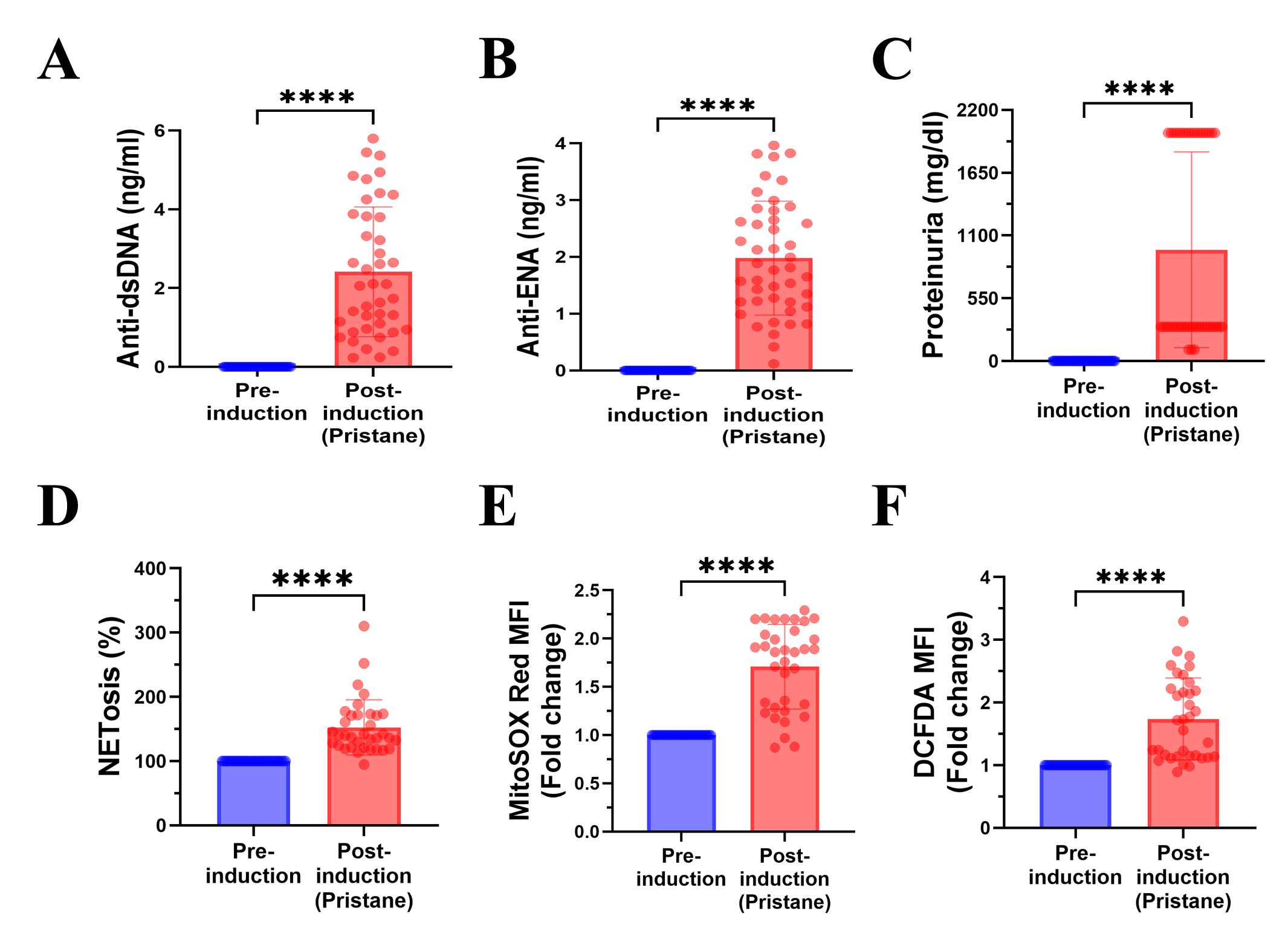

Supplement: Supplementary Figure 3 — Development of PIL mice model. Intraperitoneal injection with pristane significantly increased (A) anti-dsDNA, (B) anti-ENA, (C) urine protein (D) extracellular DNA (E) mitochondrial as well as (F) cytoplasmic ROS in a span of 35 days as compared to the pre-injected levels in mice (n=35). The bar graphs illustrate mean data from a sample of 35 mice. Error bars show mean ± SEM. p values indicate significant changes as follows: ****p < 0.0001; Student T-test. [file Image3.tif]
